# Compliance to HIV treatment monitoring guidelines can reduce laboratory costs

**DOI:** 10.4102/sajhivmed.v17i1.449

**Published:** 2016-05-31

**Authors:** Naseem Cassim, Lindi M. Coetzee, Kathryn Schnippel, Deborah K. Glencross

**Affiliations:** 1 National Health Laboratory Service, National Priority Programmes, South Africa; 2 Department of Haematology and Molecular Medicine, University of Johannesburg, South Africa; 3 Department of Haematology and Molecular Medicine, University of the Witwatersrand, South Africa; 4 Right to Care, Johannesburg, South Africa; 5 Department of Clinical Medicine, University of the Witwatersrand, South Africa

## Abstract

**Background:**

Panel tests are a predetermined group of tests commonly requested together to provide a comprehensive and conclusive diagnosis, for example, liver function test (LFT). South African HIV antiretroviral treatment (ART) guidelines recommend individual tests for toxicity monitoring over panel tests. In 2008, the National Health Laboratory Services (NHLS) request form was redesigned to list individual tests instead of panel tests and removed the ‘other tests’ box option to facilitate efficient ART laboratory monitoring.

**Objectives:**

This study aimed to demonstrate changes in laboratory expenditure, for individual and panel tests, for ART toxicity monitoring.

**Method:**

NHLS Corporate Data Warehouse (CDW) data were extracted for HIV conditional grant accounts to assess ART toxicity monitoring laboratory expenditure between 2010/2011 and 2014/2015. Data were classified based on the tests requested, as either panel (LFT or urea and electrolytes) or individual (alanine transaminase or creatinine) tests.

**Results:**

Expenditure on panel tests reduced from R340 million in 2010/2011 to R140m by 2014/2015 (reduction of R204m) and individual test expenditure increased from R34m to R76m (twofold increase). A significant reduction in LFT panel expenditure was noted, reducing from R322m in 2010/2011 to R130m in 2014/2015 (60% reduction).

**Conclusion:**

Changes in toxicity monitoring guidelines and the re-engineering of the NHLS request form successfully reduced expenditure on panel tests relative to individual tests. The introduction of order entry systems could further reduce unnecessary laboratory expenditure.

## Introduction

Diagnostic laboratory services are considered an integral part of the public health system in South Africa.^1^ Laboratory testing plays a pivotal role across the HIV continuum of care, including screening of asymptomatic individuals to identify risk for developing disease, detecting disease at the earliest stages, selecting safe and effective treatments, planning disease management strategies, monitoring treatment response throughout the course of care and identifying adverse reactions.^2^

The National Health Laboratory Service (NHLS) is the diagnostic pathology service provider to the public health sector as mandated by Act 37 of 2000.^3^ The NHLS currently provides laboratory services coverage up to 80% of the population through a national network of over 260 laboratories. Its mandate is to provide diagnostic laboratory services, research, teaching and training.^4^

Annual laboratory expenditure has increased by 45% from R3.1 billion in 2010/2011 to R4.5bn by 2013/2014.^4^ Annual state price increases during this period accounted for only 18%, the remainder being due to changes in test volumes and test mix. Total laboratory test volumes increased from 80.2 million tests in 2011/2012 to 86 million tests in 2013/2014. This sharp increase in expenditure and test volumes could be attributed to growth in the priority public health programmes, that is, HIV, TB and cervical cancer screening, as well as state price increases.^4^

Pressures on public health expenditure therefore require the application of evidence-based laboratory medicine (EBLM) that integrates clinical decision-making and laboratory investigations, to improve patient outcomes and ensure the effective use of healthcare resources.^5^ The EBLM approach includes eliminating laboratory tests with limited clinical value and introducing laboratory tests where published evidence proves their efficacy and effectiveness.^5^

For all antiretroviral treatment (ART)-related testing, the HIV and AIDS conditional grant funds are used. The conditional grant for Comprehensive Care, Management and Treatment of HIV and AIDS (CCMT) is allocated by the national government to provinces.^6^ Provincial departments of health reimburse the NHLS on a fee-for-service billing arrangement, a payment model in which the service provider is reimbursed for specific service or services provided to a patient.^7^ In this payment model, laboratory expenditure is itemised as tariff code or codes. Each test or set of tests is allocated a tariff code, for example, tariff code 02960 for the creatinine test.

Panel or profile tests are a predetermined group of diagnostic tests that are commonly requested together to provide a comprehensive and conclusive diagnosis, for example, urea and electrolytes (U&E) and liver function tests (LFT) for the assessment of renal and liver functions, respectively. Each panel consists of related individual (discrete) tests. If one of the individual tests can provide sufficient information for clinical management, replacing the more expensive panel test with specifically directed individual tests could reduce laboratory costs without negatively affecting patient outcomes or clinical interventions. However, where an LFT panel is motivated by a clinician to exclude symptoms suggestive of hepatitis, the test should be performed in line with the current ART guidelines.

ART can cause a wide range of toxicities, from low-grade intolerance that may be self-limiting to life-threatening side effects. ART toxicity can be monitored clinically, as well as by a limited number of laboratory tests. The South African ART guidelines list the ART regimens used, as well as the routine laboratory tests required for monitoring for drug toxicity.^8^ Since 2004, guidelines have recommended individual tests over panel tests. The current 2015 ART guidelines recommend alanine transaminase (ALT) testing to monitor nevirapine (NVP) toxicity and creatinine testing for tenofovir (TDF) toxicity.^9^

One of the early challenges was the availability of the ‘other tests’ option, which healthcare workers to order laboratory tests, including panels, on the CCMT request form. Since 2008, a demand-management strategy was implemented by listing only tests prescribed by the ART guidelines on the CCMT request form and removing the box for ‘other tests’, to limit the latter practice not prescribed in the ART guidelines.

## Objective

The aim of the study is to review HIV & AIDS conditional grant laboratory expenditure for ART toxicity monitoring by comparing expenditure attributable to costs of panel versus individual prescribed test ordering over a 5-year period.

## Methods

### Laboratory billing and expenditure

Conditional grant laboratory expenditure allocation is managed through a dedicated (CCMT) request form. Health facilities use the CCMT request form when tests are requested for screening and ART monitoring. Each province has either one or more ‘ZARV’ accounts on the NHLS billing systems (based on provincial requirements) to which conditional grant laboratory expenditure is allocated.

Currently, eight provinces are using conditional grants to pay for ART-related toxicity monitoring. However, it was not possible to extract conditional grant expenditure data for the KwaZulu-Natal province as they do not make use of a provincial conditional grant account. Instead, all expenditure is allocated to the health facility without the possibility of flagging CCMT-related toxicity monitoring. Laboratory expenditure data were extracted from the NHLS Corporate Data Warehouse (CDW) for ART (NVP and TDF)-related toxicity monitoring (refer to Table 1) between 2010/2011 and 2014/2015.

**TABLE 1:** Classification of conditional grant tariff codes based on the tests requested to differentiate individual and panel testing.

Antiretroviral	Panel	Panel price	Individual test	Tariff code	Individual test price
Nevirapine (NVP)	Liver Function Test (LFT)	R277.38	Alanine Transaminase (ALT)	02685	R 40.91
		-	Albumin	02700	R 36.23
		-	Aspartate Transaminane (AST)	02755	R 40.91
		-	Total Bilirubin	02780	R 31.77
		-	Direct Bilirubin	02786	R 24.19
		-	Gamma GT	03040	R 40.91
		-	Alkaline Phosphatase	03295	R 38.98
		-	Total Protein	03355	R 23.48
Tenofovir (TDF)	-	-	Creatinine	02960	R 27.32
	Urea and Electrolytes (U&E)	R76.78	-	02661	-

The data extract included the established tariff codes for each test performed,^4^ for example, 02685 for ALT. Additional variables captured from the CDW included the Province, Billable Account Number, Customer Name, Financial Period, Test Volume and Laboratory Expenditure.

Laboratory expenditure data were categorised into profiles and individual tests based on the test or tests requested and collated over the described 5-year period. For example, where an U&E test was requested (tariff code 02661), expenditure was classified as a panel test. Similarly, where an ALT test was requested (tariff code 02685), expenditure was classified as an individual test (refer to Table 1 for the categorisation criteria used to identify panels and individual tests). The individual 2014/2015 creatinine test is charged at R27.32 compared to R76.78 for the U&E panel (64% less expensive); the ALT individual test is charged R40.91 compared to R277.38 for the LFT panel (85% less expensive).

## Results

### Conditional grant laboratory expenditure on individual and panel tests

**FIGURE 1:**
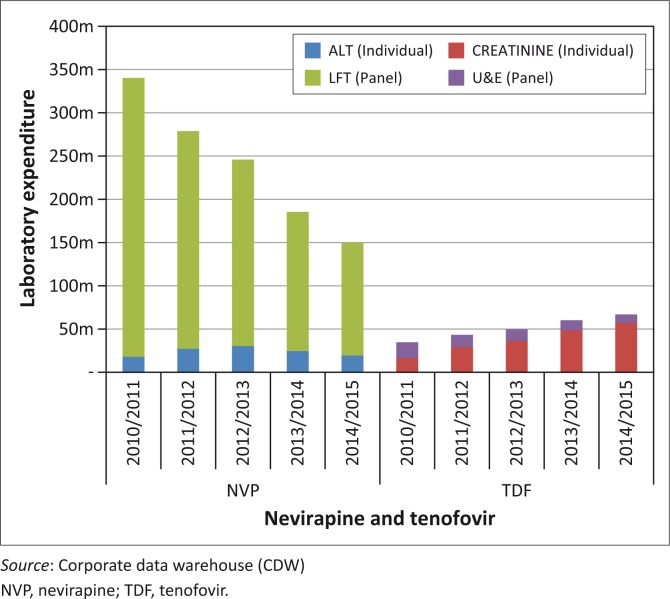
Annual laboratory expenditure for nevirapine and tenofovir toxicity monitoring of antiretroviral treatment patients 2010/2011 to 2014/2015 by panel (liver function test and urea and electrolytes) and individual testing (antiretroviral treatment and creatinine), ZAR millions.

Conditional grant laboratory expenditure on individual tests in 2010/2011 contributed 9% (R34 million) of the total expenditure for NVP and TDF toxicity monitoring. This increased each year, ultimately contributing 35% (R76m) by 2014/2015 (Figure 1). In 2010/2011, expenditure on panel tests comprised 91% (R340m) of toxicity monitoring expenditure, reducing by over 26% to 65% by 2014/2015 (R140m). The overall expenditure on panel tests reduced from R340m in 2010/2011 to R140m by 2014/2015. Similarly, expenditure on individual tests increased from R34m to R76m (twofold increase). A total reduction of R200m in laboratory expenditure incurred through panel testing was achieved in 2014/2015.

There was a significant reduction in NVP-related panel testing, reducing from R322m in 2010/2011 to R130m in 2014/2015 (reduction of R192m) (Figure 1). With the introduction of TDF in 2010, TDF-associated individual test expenditure increased from R16m in 2010/2011 to R56m in 2014/2015 (more than a three-fold increase). A reduction in laboratory expenditure of R192m was achieved through reduced NVP-related panel testing in 2014/2015.

### Percentage of provincial conditional grant laboratory expenditure on panel tests

**FIGURE 2:**
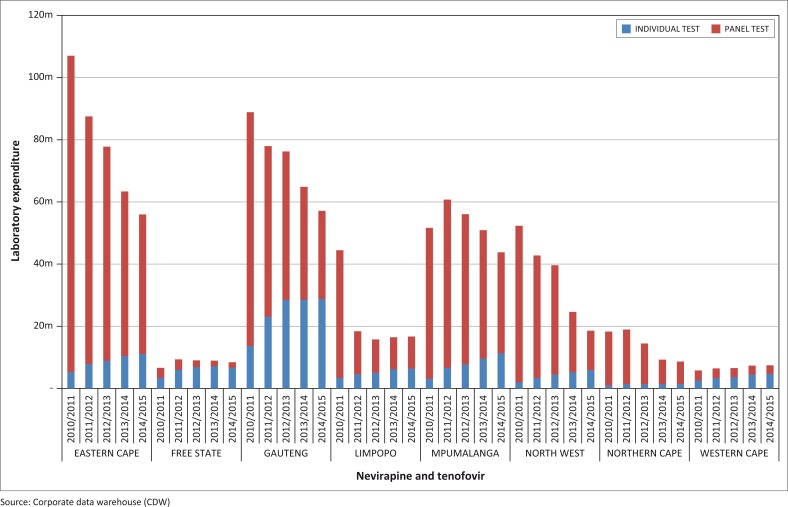
Annual laboratory expenditure for Nevirapine and Tenofovir toxicity monitoring of antiretroviral treatment patients 2010/2011 to 2014/2015, by individual or panel category, ZAR millions.

Overall, the Free State province reported the lowest percentage of expenditure on panel testing reducing from 48% in 2010/2011 to 21% by 2014/2015 (Figure 2). The Western Cape province reduced from 56% in 2010/2011 to 36% by 2014/2015. The Gauteng and Limpopo provinces also made significant progress in reducing panel tests to below the national average by 2014/2015. The Gauteng province reduced the percentage of laboratory expenditure on panel tests from 85% in 2010/2011 to 70% by 2011/2012 (15 percentage point change). Similarly, the Limpopo province reduced from 92% in 2010/2011 to 75% by 2011/2012 (17 percentage point change). By 2014/2015, the Gauteng province had reduced expenditure on panel tests to 50%, whilst Limpopo had decreased to 62%. The overall reduction in panel testing between 2010/2011 and 2014/2015 varied between 12% (Northern Cape) and 35% (Gauteng) with a median of 23%, with averaged reduction in laboratory expenditure of R200m attributable to guideline adherence in 2014/2015.

## Discussion

Laboratory expenditure on ART toxicity monitoring (NVP and TDF) reported a significant reduction in the relative proportion of panel testing compared to individual tests between 2010/2011 and 2014/2015, from 91% panel testing to 65% panel testing. A significant reduction in the number of LFT panels ordered, decreasing laboratory expenditure from R322m in 2010/2011 to R130m in 2014/2015, aligns with change in first-line ART regimens during the period away from NVP. Thus, laboratory services demand-management facilitating ART guidelines, together with the request form refinement, were effective in reducing laboratory tests requests and hence, expenditure.

However, despite ART guidelines recommending use of the individual tests and with the demand-management request form in place, there is still substantial panel test laboratory expenditure present in 2014/2015 (R140m), especially for NVP monitoring (R130m), leaving room for additional savings.

### Recommendations to achieve further efficiencies

To further reduce laboratory expenditure of panel tests and to facilitate appropriate guideline-based testing, two options are available. The first alternative involves stopping inappropriately requested tests before they reach the laboratory network, unless the panel test is motivated for by a consultant.^10^ This will enable clinicians to request the panel test in patients with symptoms suggestive of hepatitis. These preventative strategies involve a focus on education for clinicians and nurses on appropriate test ordering based on the latest ART guidelines.^10^ These interventions include healthcare worker training on appropriate test requesting and the inclusion of interpretative comments on laboratory test results,^10^ for example, ‘An LFT is not indicated for patients on ART, please request an ALT as stipulated by the current ART guidelines’.

The second intervention to manage appropriate test requesting involves the development of Order Entry (OE) applications on existing electronic health records and hospital information systems, to flag inappropriate test requests before venesection commences at the health facility. This is the most effective option and alerts (and educates) the healthcare worker requesting an inappropriate test, upfront, by offering more appropriate and cost-effective tests.

Westbrook et al. reported that one of the main advantages of computerised OE systems is the ease of extracting and reviewing the impact of laboratory demand-management strategies by using real-time data to feedback to clinicians and nurses.^11^ To describe this further, computerised OE refers to an application that enables healthcare workers to order laboratory tests using a computer system at or near patient-care areas.^12^ Additionally, OE can provide a platform that streamlines the logistical processes before the samples get to the laboratory, standardises ordering of laboratory tests, promotes adherence to guidelines and delivers decision support alerts.^12^ Additional benefits of OE include the reduction of duplicate test orders for same patient.^12,13^ The removal of panel tests on the OE screen itself can reduce panel orders and increase individual test orders.

OE systems may be confused with electronic gate keeping (EGK), which in comparison, is a rule-based mechanism employed on laboratory information systems to reject tests based on agreed criteria when they reach the laboratory. The aim of EGK is to prevent or minimise irrational and wasteful use of laboratory services. The challenge with EGK is that tests are rejected at the laboratory, initially unbeknown to the attending clinician, whereas with OE the decision support alerts are generated at the health facility, enabling the healthcare worker to immediately respond. OE also supports the electronic delivery of laboratory results for integration into the patient’s record in the hospital information system.^12^ OE also saves wasted expenditure by removing the cost associated with pulling the sample and sending it to the laboratory in the first place (approximately R2.58 for the vacutainer tube, specimen plastic bag and request form). Implementing OE systems in South Africa will require adequate information technology (IT) infrastructure, which is currently lacking.

The combination of these interventions could act to unlock additional reductions in laboratory expenditure on toxicity monitoring and meaningfully reduce public health expenditure on providing ART services.

### Limitations

Due to the exclusion of the KwaZulu-Natal province, this study is not representative of national toxicity monitoring expenditure. From the data extract, we were unable to differentiate test orders from hospitals and primary healthcare clinics to assess differences in laboratory expenditure patterns by level of care. Additionally, it was not possible to differentiate between routine toxicity monitoring versus testing following an adverse event. However, hospital expenditure is funded through the provincial equitable share and not the HIV conditional grant, and therefore, this bias is not likely to be substantial.

## Conclusion

Although there have been significant cost reductions in panel testing reported here following some fairly simple interventions, widespread use of these interventions is necessary to fully exclude unnecessary laboratory expenditure and maximise cost-efficiency in delivering laboratory services required for monitoring ART toxicity. The introduction of an OE system could play a significant role in this regard to reduce inappropriate laboratory test requests and public health expenditure in South Africa. Additionally, the introduction of OE would improve the appropriate utilisation of laboratory services across all disciplines to further reduce public health expenditure.
